# Vaccination of Cattle with the N Terminus of LppQ of Mycoplasma mycoides subsp. mycoides Results in Type III Immune Complex Disease upon Experimental Infection

**DOI:** 10.1128/IAI.00003-15

**Published:** 2015-04-15

**Authors:** Musa Mulongo, Joachim Frey, Ken Smith, Christian Schnier, Hezron Wesonga, Jan Naessens, Declan McKeever

**Affiliations:** a International Livestock Research Institute, Nairobi, Kenya; b Department of Pathology and Pathogen Biology, Royal Veterinary College, Hatfield, Hertfordshire, United Kingdom; c Department of Veterinary Bacteriology, University of Berne, Berne, Switzerland; d Moredun Research Institute, Penicuik, Midlothian, Scotland, United Kingdom; e National Veterinary Research Center, Muguga, Kikuyu, Kenya

## Abstract

Contagious bovine pleuropneumonia (CBPP) is a serious respiratory disease of cattle caused by Mycoplasma mycoides subsp. mycoides. Current vaccines against CBPP induce short-lived immunity and can cause severe postvaccine reactions. Previous studies have identified the N terminus of the transmembrane lipoprotein Q (LppQ-N′) of M. mycoides subsp. mycoides as the major antigen and a possible virulence factor. We therefore immunized cattle with purified recombinant LppQ-N′ formulated in Freund's adjuvant and challenged them with M. mycoides subsp. mycoides. Vaccinated animals showed a strong seroconversion to LppQ, but they exhibited significantly enhanced postchallenge glomerulonephritis compared to the placebo group (*P* = 0.021). Glomerulonephritis was characterized by features that suggested the development of antigen-antibody immune complexes. Clinical signs and gross pathological scores did not significantly differ between vaccinated and placebo groups. These findings reveal for the first time the pathogenesis of enhanced disease as a result of antibodies against LppQ during challenge and also argue against inclusion of LppQ-N′ in a future subunit vaccine for CBPP.

## INTRODUCTION

Contagious bovine pleuropneumonia (CBPP) is a serious respiratory disease of cattle characterized by coughing, fever, and respiratory distress and causing major economic losses, particularly in sub-Saharan Africa (1–3). Whereas CBPP has been controlled in a number of developed countries through a combination of coordinated surveillance, eradication of whole infected herds, and quarantine (4), these methods are not effective in developing countries due to geopolitical, economic, and cultural considerations (3, 5, 6). Vaccination is therefore considered the most appropriate control measure for CBPP in sub-Saharan Africa. However, the most widely deployed vaccine against CBPP in sub-Saharan Africa, as recommended by the Food and Agricultural Organization (FAO), is the live attenuated Mycoplasma mycoides subsp. mycoides vaccine strain T1/44, which induces only short-lived immunity and causes severe postvaccine skin lesions in a proportion of vaccinates (7). In addition, there is evidence that that CBPP-associated lung damage has a significant immunological component (8). Development of an improved vaccine for CBPP therefore requires careful evaluation of antigen candidates used for a vaccine and exclusion of those components that induce enhancement of the disease.

Surface lipoproteins have been identified as potential triggers of the massive inflammatory reaction that is caused by M. mycoides subsp. mycoides and other mycoplasmal infections. Whereas the role of mycoplasma lipoproteins in induction of proinflammatory cytokines has been demonstrated (9, 10), data as to the precise role of these proteins in the outcome of disease are limited. The M. mycoides subsp. mycoides transmembrane lipoprotein Q (LppQ) induces strong, early, and persistent antibody responses in cattle undergoing natural CBPP infection (11). This led to the development of a CBPP diagnostic enzyme-linked immunosorbent assay (ELISA) based on the membrane-exposed N-terminal fragment of LppQ (LppQ-N′) (12). Nicholas et al. conducted a preliminary study to determine the vaccine efficacy of this determinant (13). In that study, 4 cattle were vaccinated with recombinant LppQ-N′ packaged in immunostimulating complexes (ISCOMs) and boosted 6 weeks later, before being placed, along with 4 unvaccinated control animals, in contact with cattle that had been infected with CBPP by intrabronchial instillation. After 4 months, 2 vaccinated animals died, coincident with an increase in M. mycoides subsp. mycoides-specific antibody levels as measured by competition ELISA (C-ELISA) (13) and complement fixation test (CFT). In addition, 3 out of 4 control unvaccinated animals died of CBPP. Postmortem observations indicated that the LppQ-N′-vaccinated group had enhanced disease, as revealed by more extensive lung lesions and fibrin deposition as well as the presence of more abundant pleural fluid, compared to that in the placebo group. However, despite these indications, no details on the pathological outcome in this limited number of animals were given.

Therefore, we conducted a vaccine trial using LppQ-N′ formulated in Freund's adjuvant and evaluated the clinical, pathological, and immunological outcomes after challenge with an infective dose of M. mycoides subsp. mycoides. Based on our observations, we describe a novel mechanism of CBPP pathogenesis and discuss the suitability of LppQ-N′ as a vaccine candidate against CBPP.

## MATERIALS AND METHODS

### Expression and purification of recombinant LppQ-N′.

**FIG 1:**
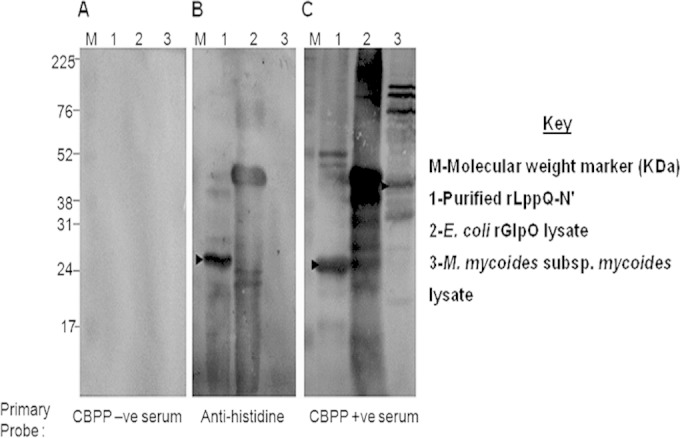
Recombinant LppQ-N′ reacts with CBPP immune sera. Immunoblots were probed with CBPP-negative bovine serum (1:100) (A), antihistidine MAb (1:4,000) (B) and CBPP-positive serum (1:100) (C). Arrows indicate the rLppQ-N′ molecule as well as the native protein in M. mycoides subsp. mycoides lysate. Recombinant histidine-tagged GlpO was used as a positive control, hence causing the positive signals in panels B and C. The high background in panel C reflects cross-reacting antibodies to E. coli proteins in unpurified rGlpO lysate.

The recombinant polyhistidine-tailed 26.5-kDa N terminus of the LppQ protein was expressed and purified from transformed Escherichia coli BL21(DE3) cells and prepared as described previously (11). Production of the LppQ-N′ fusion protein was induced by the addition of 1 mM isopropyl-β-d-1-thiogalactopyronaside (IPTG) at mid-exponential phase and incubation for a further 3 h. Following induction, the fusion protein was purified from cell extracts dissolved with 8 M urea using Ni^2+^ chelate affinity chromatography (Qiagen) according to the manufacturer's instructions. The bound fusion proteins were eluted at 250 mM imidazole in sodium phosphate buffer (20 mM NaH_2_PO_4_, 0.5 M NaCl, 8 M urea, pH 8). Following elution, the fusion protein was dialyzed against 50 mM phosphate buffer–300 mM NaCl (pH 7.4) and quantified using the test tube method of the Coomassie (Bradford) protein assay kit (Pierce, Rockford, IL, USA) as described by the manufacturer. Typically, a 100-ml culture of E. coli yielded 2 to 4 mg of the 26-kDa LppQ-N′ protein in the 200 mM imidazole fraction. The identity of LppQ-N′ was confirmed by immunoblotting using antihistidine monoclonal antibody (MAb) (Qiagen) and bovine CBPP recovery serum (Fig. 1). In this blot, unpurified lysate containing the histidine-tagged recombinant l-α-glycerol-3-phosphate oxidase (rGlpO) of M. mycoides subsp. mycoides was used as an additional positive control for LppQ-N′.

### *In vitro* culture and quantification of M. mycoides subsp. mycoides.

Lyophilized aliquots of the virulent M. mycoides subsp. mycoides strain B237, which originated from an acute case of CBPP in central Kenya (14), were reconstituted in prewarmed (37°C) Gourlay medium (15) and propagated as described previously (16). M. mycoides subsp. mycoides for challenge infection was quantified on the basis of color-changing units (CCU) per milliliter using the method of Spearman and Karber (17) as described previously (16). M. mycoides subsp. mycoides was considered suitable for inoculation on the third day of passage if the culture appeared filamentous and was at pH 6.5.

### Vaccination and experimental infection of cattle.

Vaccination of cattle was conducted in compliance with the United Kingdom Animals (Scientific) Procedures Act (1986) and was approved by the Animal Experimentation Ethics Committees of both the National Veterinary Research Center, Muguga (Nairobi, Kenya), and the Moredun Research Institute (Scotland, United Kingdom). This was a randomized double-blind vaccine trial where 14 1-year-old Zebu steers from a CBPP-free zone that tested seronegative by CFT were assigned unique identifiers and randomized into 2 equal groups using an MS Excel spreadsheet. One group was inoculated subcutaneously with 200 μg of recombinant LppQ-N′ in 1 ml of phosphate-buffered saline (PBS) (pH 7.4) emulsified in an equal volume of complete Freund's adjuvant (CFA). Cattle were boosted after 4 weeks and 6 weeks with the same quantity of antigen emulsified in incomplete Freund's adjuvant (IFA) (Sigma). The same number of animals in the placebo group was similarly inoculated with 2 ml of PBS-CFA emulsion and boosted with PBS-IFA.

For challenge infection, cattle were sedated by subcutaneous injection of 2 ml undiluted xylazine (Bayer AG, Leverkusen, Germany) and inoculated via an endobronchial tube with 60 ml of culture (at exponential phase and pH 6.5) containing ∼10^8^ CCU/ml of M. mycoides subsp. mycoides. This inoculum was immediately followed by 30 ml of PBS (pH 7.4) and 15 ml of prewarmed 10% low-melting-point agar. Rectal temperatures were measured daily thereafter and clinical signs monitored throughout the period of the experiment. Animals showing continuous fever for 10 days were euthanized on ethical grounds, while all other survivors were euthanized on the 35th day after challenge infection. All animals were bled for preparation of serum before challenge and prior to euthanasia. During the entire period of the study, all animals were housed together. The identity of animals in each group was kept secret until the end of the trial. To ensure uniformity and blinding, the challenge infection and clinical monitoring were conducted by a veterinarian who did not share information with the postmorterm pathologist.

### Postmorterm lung pathological scoring.

A pathology score for each animal at postmorterm was calculated using a modification of the scoring system described by Hudson and Turner (18). Briefly, a lesion score of 1 was assigned to a resolving lesion with only fibrous tags or pleural fibrous adhesion lesion score, a score of 2 was added to the lesion score if mycoplasma was isolated from the lung lesion, and the total was multiplied by a factor determined by the diameter of the lesion (1 for ≤5 cm, 2 for ≤20 cm but >5 cm, and 3 for >20 cm). Hence, the maximum pathology score for each animal was (2 + 2)3 = 12.

### Isolation of M. mycoides subsp. mycoides from experimental cattle.

Lung tissue from areas with lesions and those appearing grossly normal was excised aseptically, immersed in bottles containing 50 ml prewarmed Gourlay medium (with phenol red indicator, which turns yellow as the medium pH changes due to bacterial metabolism or contamination with other microbes), and incubated at 37°C in a humidified incubator with 5% CO_2_. The cultures were examined daily for growth and/or contamination. On the third day, after change of color from red to yellow, cultures were streaked on prewarmed tryptose agar plates (formulated as for Gourlay medium but containing 20 g/liter agar [Difco]), which were sealed and incubated at 37°C for 10 days. Four dilutions (1:10, 1:20, 1:30, and 1:40) of each broth culture were also prepared in fresh medium and examined daily for growth. M. mycoides subsp. mycoides was considered to be present if all dilutions of broth cultures changed from orange to yellow within 14 days and when corresponding agar plates showed mycoplasmal morphological features, including the typical “fried egg” appearance characteristic of M. mycoides subsp. mycoides colonies.

### Immunoblotting and complement fixation test.

Immunoblots were prepared as described previously (19) with some modifications. Briefly, proteins were transferred to a nitrocellulose (NC) membrane with a pore size of 0.45 μm (Protran Whatman Gmbh, Germany) using the Hoefer electroblotting system (Serva Electrophoresis GmBH, Heidelberg, Germany) under a constant voltage of 20 V overnight at 4°C. Membranes were blocked with 5% bovine serum albumin (BSA) in PBS-T (Tris-buffered saline, 0.05% Tween 20) for 1 h and then washed in PBS-T before being incubated for 1 h with primary antibody diluted 1/100 in PBS-T and then washed and incubated for 1 h with horseradish peroxidase-conjugated goat anti-bovine IgG (Sigma). Bands were visualized by incubation in 30% diaminobenzidine (DAB) and 1% H_2_O_2_ in PBS until sufficient resolution was achieved, whereupon the reaction was stopped by the addition of 1 mM HCl.

The CBPP CFT kit (CIRAD, Montpellier, France) was used to detect antibodies against M. mycoides subsp. mycoides following challenge infection. Essentially, 25 μl of test sample serum diluted in kit Veronal buffer was distributed in 96-well round bottom plates (Costar Corning, MA, USA) before addition of M. mycoides subsp. mycoides antigen and diluted kit guinea pig complement. The mixture was vigorously shaken and incubated with periodic shaking for 30 min at 37°C before addition of the hemolytic system (3% sheep red blood cells [SRBC] and hyperimmune anti-SRBC rabbit serum) and further incubation for 30 min at 37°C with periodic shaking. The plates were then centrifuged at 125 × *g* for 2 min, and the extent of hemolysis was determined by examination over a mirror. Test serum was considered positive for CBPP antibodies if complete inhibition of hemolysis was observed at a dilution of 1/10.

### Processing and staining of tissues for light microscopy.

Formalin-fixed tissues were trimmed, placed in labeled cassettes, and automatically dehydrated (Shandon Hypercenter XP system; Global Medical Instrumentation [GMI], Ramsey, MN, USA). The tissues were embedded in wax using an automatic tissue processor (Triangle Biomedical Sciences Inc., Durham NC, USA). Four 4-μm sections were cut and floated on the surface of a preheated (45 to 55°C) water bath before transfer to a microscope slide. Tissue sections were rehydrated by sequential immersion in xylene (3 times for 3 min) and ethanol (3 times for 3 min) and 3 washes in 95% ethanol, 80% ethanol, and deionized water, respectively. Sections were dipped in Gills hematoxylin (VWR) for 10 min before washing with water, counterstaining with 1% eosin (VWR) for 1 min, and immersion in 1% acid ethanol. Sections were washed in ethanol and xylene and then coverslipped using Histomount mounting solution (Vector Laboratories, Peterbrough, United Kingdom).

### Immunohistochemistry.

Four 4-μm sections were sequentially immersed in xylene, then immediately transferred to ethanol, and finally placed in a water bath for 15 min. Antigen retrieval was achieved by immersion of sections in preheated citrate buffer (0.25% trisodium citrate hydrate and 0.025% citric acid in deionized water, pH 6.1). The sections were then heated in a microwave oven (800 W for 10 min), cooled in citrate buffer, and rehydrated. Immunohistochemistry was carried out using the Novolink polymer detection system kit (Leica Microsystems, Newcastle, United Kingdom) according to the manufacturer's instructions. A monoclonal antibody (MAb) raised against the membrane-located l-α-glycerol-3-phosphate (GlpO) of M. mycoides subsp. mycoides (produced in-house at the International Livestock Research Institute [ILRI]), at the optimal dilution of 1:500, was used as primary antibody. The sections were dehydrated and visualized after mounting with Histomount (Vector).

### Statistical analysis.

Nonparametric tests were used to analyze outcome variables. The two-tailed Mann-Whitney test was used to analyze lung pathological scores and duration of fever, while Fisher's exact test was used to analyze kidney pathology, lung pathology (vasculitis), and clinical signs of coughing and respiratory distress. Postchallenge survival was analyzed by the log rank (Mantel-Cox) test. All statistical analyses were performed using Prism version 5.04 for Windows (GraphPad Software, San Diego, CA, USA).

## RESULTS

### Antibody responses.

**FIG 2:**
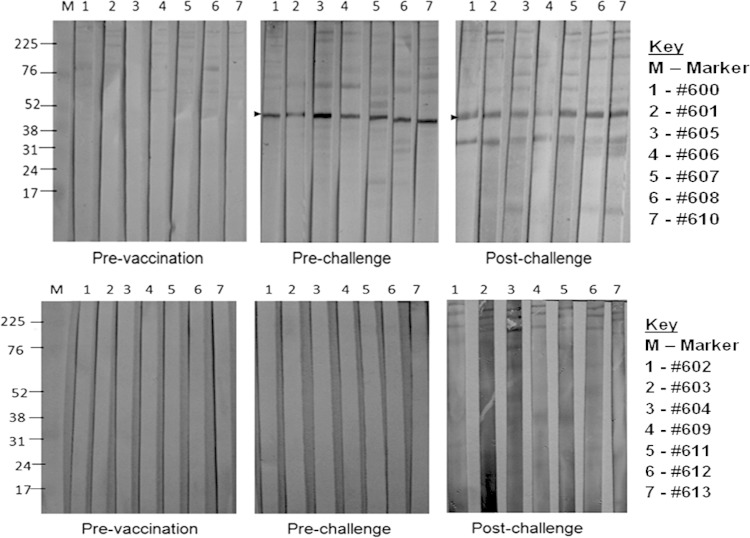
Vaccination of cattle with rLppQ-N′ induces antibody responses to native full-length LppQ. Top panels, LppQ-vaccinated group; bottom panels, placebo group. For each animal, serum was taken before vaccination, 1 day before challenge, and on the day of necropsy. Each lane contains M. mycoides subsp. mycoides total lysate reacted with bovine serum at a dilution of 1/100. Anti-LppQ reactions are revealed as a band corresponding to 48 kDa representing the full-size LppQ.

To evaluate antibody responses to LppQ-N′, total M. mycoides subsp. mycoides lysate was probed in immunoblots with sera taken from individual animals before vaccination, 1 day before challenge, and on the day of euthanasia (Fig. 2). Vaccination with rLppQ-N′ induced antibodies to LppQ in all immunized animals before challenge (Fig. 2, top panels), with a number of apparently nonspecific determinants of various sizes presenting with weaker signals. These weaker determinants were also present in samples taken after challenge but prior to euthanasia, although the dominant response remained toward LppQ and an additional protein of approximately 34 kDa. In the placebo group, no specific LppQ responses were detected before or after challenge (Fig. 2, bottom panels). We also measured complement-fixing antibodies in sera from all animals 1 day before challenge and on the respective day of euthanasia for each animal (Table 1). No animal in either group showed positive CFT titers before challenge (data not shown). However, all animals tested positive for CFT antibodies prior to euthanasia, with the exception of one (animal 609) in the placebo group. Titers ranged from 1/10 to 1/80 and were typically higher in the vaccinated group (Table 1).

**TABLE 1:** Complement-fixing antibody titers to total M. mycoides subsp. mycoides antigen in vaccinated and placebo group animals on the respective day of euthanasia

Group	Animal	CFT titer^*a*^
LppQ-N′	600	1/40
	601	1/80
	605	1/20
	606	1/40
	607	1/20
	608	1/40
	610	1/80
Placebo	602	1/20
	603	1/10
	604	1/10
	609	0
	611	1/20
	612	1/10
	613	1/10

a Test serum was considered positive for CBPP antibodies if complete inhibition of hemolysis was observed at a dilution of 1/10. Values are the highest dilution at which inhibition of hemolysis was observed.

### Clinical signs, gross pathological findings, and isolation of M. mycoides subsp. mycoides.

All study animals were monitored for the following 3 key clinical signs of acute and subacute CBPP: presence of coughing, presence of respiratory distress, and duration of fever. No statistical differences (coughing, *P* = 1.0; respiratory distress, *P* = 0.19; median duration of fever, *P* = 0.5) between the vaccinated and placebo groups were observed for all these parameters.

**FIG 3:**
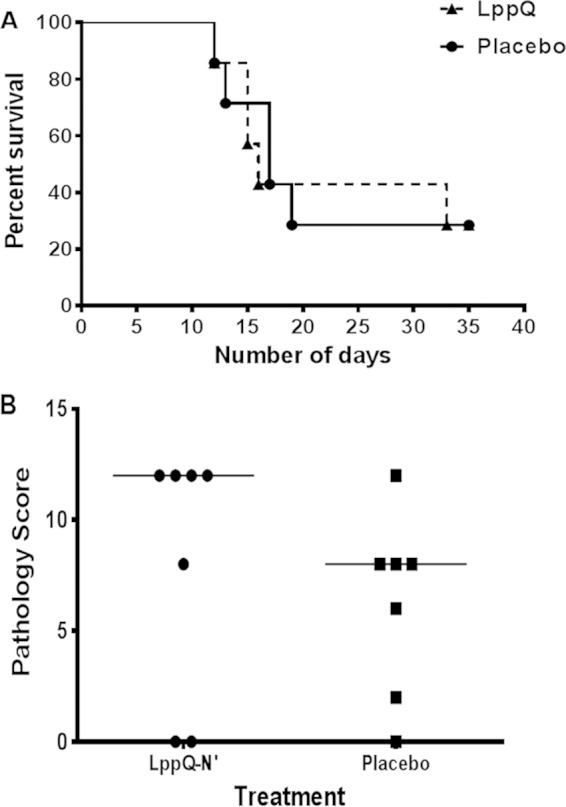
Vaccination of cattle with LppQ-N′ has no effect on the postchallenge survival rate, although it seems to be associated with higher pathological scores. (A) Survival curve displaying the rate of survival of LppQ-N′-vaccinated and placebo groups following challenge infection. Animals were euthanized after 10 days of continuous fever or at the end of the trial (35th day after challenge). (B) Gross pathological scores of vaccinated and placebo groups. The combined pathological scoring focused on size and appearance of lung lesions and isolation of M. mycoides subsp. mycoides, with a score of 12 as the most severe and 0 as absence of lung lesion.

Two cattle (28.6%) in each group survived until being euthanized on the 35th day after challenge (Fig. 3A). Of these 4 survivors, only one animal, from the placebo group, was found to have CBPP lung lesions. Using the log rank (Mantel-Cox) test, there was no significant difference between the experimental and placebo groups in the proportions that survived challenge infection (*P* = 0.939). Postmorterm pathological scores were determined as described in Materials and Methods and are shown in Fig. 3B. Pathological scores did not significantly differ (*P* = 0.39) between the vaccinated and placebo groups using the two-tailed Mann-Whitney test. Lung lesions were found in five vaccinated animals (71.4%), and in six placebo group animals (85.7%). In areas with lesions, lung tissue was swollen (up to 2 to 3 times the original size), edematous, and consolidated. On excision, the lesions exhibited red hepatization, thickened interlobular septa, and a marbled appearance. All cattle with lung lesions showed pathological features of fibrinous bronchopneumonia with pleuritis that are typical of acute and subacute CBPP. Most affected pleural surfaces were covered by scanty to moderate deposits of fibrin. In all cattle with lesions, the mediastinal lymph nodes draining the caudal lobes were enlarged.

**FIG 4:**
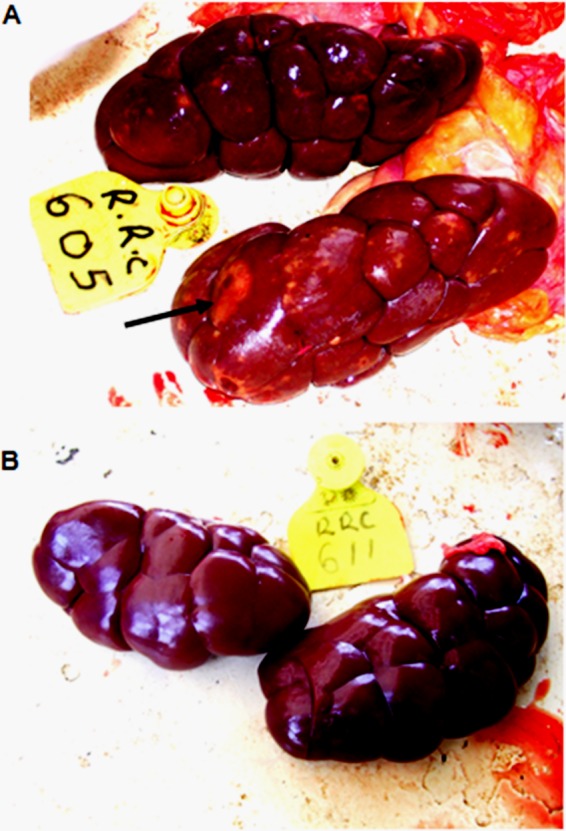
Experimental infection of LppQ-N′-vaccinated cattle results in gross kidney lesions. Photomicrographs of kidney appearance are shown. (A) LppQ-N′-vaccinated animal 607; (B) placebo group animal 609. Note the scattered lesions on the kidney of the vaccinated animal; the largest lesion is shown by the arrow. The placebo group animal shows a grossly normal kidney.

The most pronounced difference between the vaccinated and placebo groups was the presence of scattered pale kidney lesions in 5 (71.4%) animals of the former group but in none of the latter group (Fig. 4). This difference was statistically significant using Fisher's exact test (*P* = 0.021). Notably, all 5 vaccinated animals that had lung lesions also displayed kidney lesions. The pale foci were scattered on the visceral surface and confined to the cortex. Each lesion varied between 0.5 cm and 3.0 cm in diameter (Fig. 4A).

### Histological and immunohistological findings.

**FIG 5:**
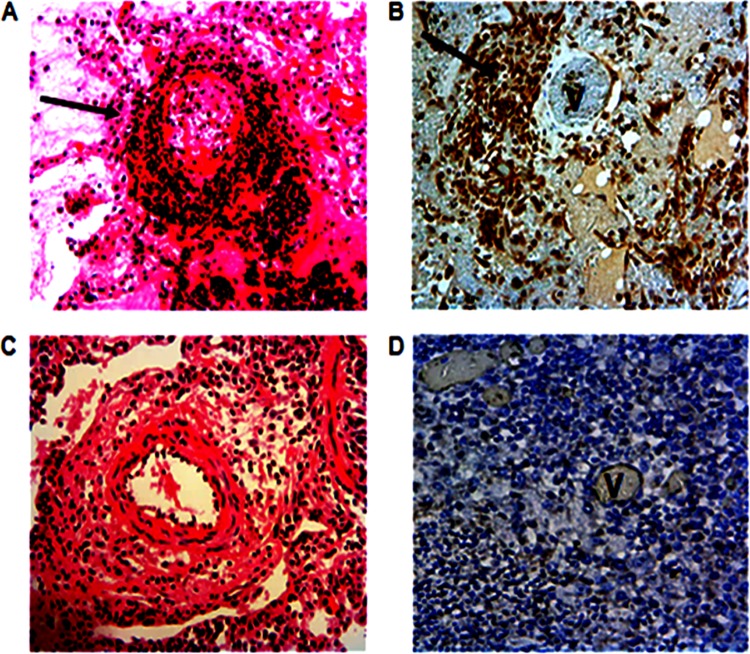
Experimental infection of LppQ-N′-vaccinated cattle results in pulmonary vasculitis that corresponds with the presence and distribution of M. mycoides subsp. mycoides antigen. (A and C) H&E-stained sections of pulmonary blood vessels from vaccinated animal 605 (A) and placebo group animal 609 (C). Dense perivascular invasion by lymphocytes and occasional macrophages is evident in the section from animal 605 and is marked by an arrow. Magnification, ×300. (B and D) IHC staining for M. mycoides subsp. mycoides antigen in pulmonary blood vessels from vaccinated animal 608 (B) and placebo group animal 603 (D). The arrow in panel B shows M. mycoides subsp. mycoides antigen concentrated around the vascular endothelium. M. mycoides subsp. mycoides antigen is not present in the endothelium of animal 603. Magnification, ×300.

Hematoxylin and eosin (H&E) staining of lung lesions obtained from both the vaccinated and placebo groups showed large irregular areas of necrosis surrounded by large numbers of lymphocytes and neutrophils and occasional macrophages in a dense fibrous stroma. Some lesions were flooded with a proteinaceous exudate and were edematous. There was marked thickening of the interlobular septa, and alveolar walls were infiltrated with macrophages and giant cells. Fibrinoid necrosis of blood vessel walls and intramural and perivascular invasion with neutrophils and lymphocytes (leukocytoclastic vasculitis and cuffing) were observed in 4 vaccinated cattle that also had lung lesions (Fig. 5A and B) but were absent in unvaccinated animals (Fig. 5C and D) except for 1. Nonetheless, this difference was not significant using Fisher's exact test (*P* = 0.26). In vaccinated cattle, immunohistochemical staining with anti-GlpO MAb revealed a strong association between vasculitis and presence of M. mycoides subsp. mycoides antigen around the vascular endothelium (Fig. 5A and B).

**FIG 6:**
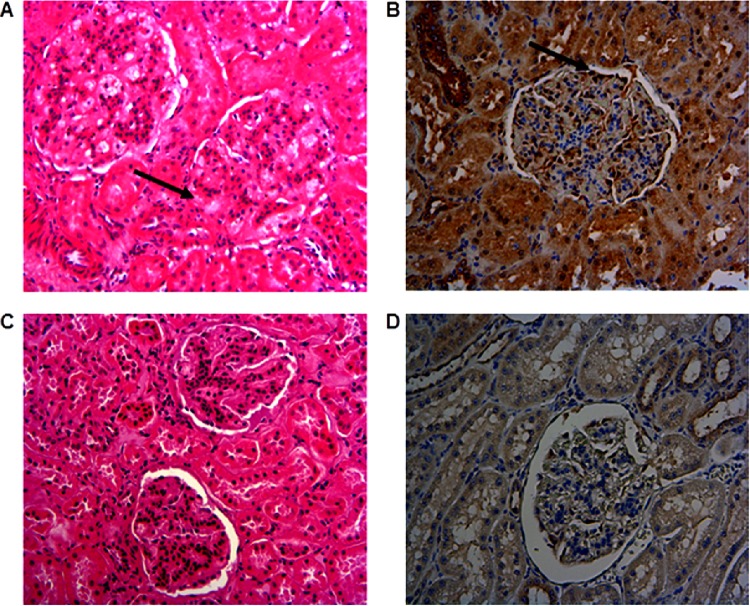
Experimental infection of LppQ-N′-vaccinated cattle results in glomerulonephritis that is associated with the presence of M. mycoides subsp. mycoides antigen. (A and C) H&E-stained sections of glomeruli. (A) The glomerular basement of vaccinated animal 605 is thickened (arrow), and scattered lymphocytic aggregates can be seen in the interstitium. (C) These changes are absent in the glomerulus of placebo group animal 611. Magnification, ×350. (B and D) IHC staining for M. mycoides subsp. mycoides antigen in glomeruli. (B) Glomerulus of vaccinated animal 608, showing M. mycoides subsp. mycoides antigen along the glomerular basement membrane (arrow). (D) Glomerulus of placebo group animal 611 without M. mycoides subsp. mycoides antigen.

Hematoxylin and eosin staining and immunohistochemical staining of kidney sections are shown in Fig. 6. In kidney sections of vaccinated cattle, widespread mesangial thickening of the glomerular basement membrane was observed, accompanied by scattered small interstitial lymphoplasmacytic aggregates and areas of fibrosis around the glomerulus (Fig. 6B). In contrast, kidney tissues from the placebo group had either no detectable abnormality or only mild mesangial thickening (Fig. 6C). Immunohistochemical staining of kidney sections from vaccinated cattle with kidney lesions revealed the presence of M. mycoides subsp. mycoides antigen along the glomerular basement membrane (Fig. 6B). However, no M. mycoides subsp. mycoides antigen was observed in unvaccinated cattle (Fig. 6D).

## DISCUSSION

The transmembrane lipoprotein LppQ of M. mycoides subsp. mycoides is known to induce strong, specific, and persistent antibody responses in infected cattle (11), which makes it an attractive candidate for serological diagnosis of CBPP) (12). However, its utility as a vaccine has been challenged by a preliminary study that revealed exacerbation of disease following challenge of 4 cattle vaccinated with recombinant LppQ-N′ formulated in ISCOMs (13). We intended to clarify this situation by undertaking a robust evaluation of the responses of cattle to immunization with LppQ-N′ and the outcome of their challenge with M. mycoides subsp. mycoides.

All animals seroconverted to LppQ following immunization, as revealed by immunoblotting of pre- and postchallenge sera. Interestingly, only one additional determinant, of 34 kDa, was evident in immunoblots probed with postchallenge sera (Fig. 2, to panels), The reasons behind this narrow response to challenge are unclear, as is the basis of the virtual absence of a response in the animals in the placebo group following challenge. This may reflect the timing of sampling; both CFT and LppQ ELISA titers peak at approximately 50 days following infection and are low to undetectable at day 35 (12). It is possible that the additional reaction to the 34 kDa antigen of the M. mycoides subsp. mycoides band seen in the LppQ-N′-immunized cattle after challenge with M. mycoides subsp. mycoides arose from enhanced presentation of M. mycoides subsp. mycoides antigen as a result of opsonization of the organism with anti-LppQ antibodies.

Since no detectable clinical changes were observed in cattle after vaccination, it appears that LppQ-N′ alone does not induce a reaction of clinical relevance. However, postchallenge observations indicated that compared to the placebo group, the vaccinated group had a more severe pathological outcome, as evidenced by lesions of glomerulonephritis (Fig. 4A), and a tendency toward higher pathological scores and occurrence of vasculitis, although the latter two did not attain statistical significance. The small number of animals in the study rendered it difficult to demonstrate significant clinical and pathological differences between vaccinated and placebo group animals, except for the statistically significant presence of kidney lesions in the former. We believe that an experimental study with adequate power may confirm our observations. Considering that CBPP pathogenesis involves, at least in part, aberrant host immune responses to the pathogen (20, 21), it is reasonable to conclude that this adverse pathological outcome was mediated either wholly or in part by anti-LppQ-N′ antibodies and their interaction with the organism. The vasculitis and glomerulonephritis observed in vaccinated animals are consistent with immune complex disease and type 3 hypersensitivity (Arthus reaction). An important drawback to the current study is our inability to establish an ELISA to quantify anti-LppQ-N′ antibodies. Such an assay would have been useful to demonstrate whether high antibody titers to LppQ-N′ correspond with the occurrence of immune complexes.

Histological changes observed in the lungs of both vaccinated and unvaccinated cattle are indicative of the massive inflammation that is known to be triggered by M. mycoides subsp. mycoides invasion (21). Unlike animals in the placebo group, the majority of vaccinated animals showed fibrinoid necrosis of blood vessel walls with intramural and perivascular infiltration by neutrophils, lymphocytes, and occasional macrophages (Fig. 5A), all of which are features of vasculitis. Indeed, in addition to infiltration of the lung parenchyma adjacent to necrotic areas in these animals, inflammatory lesions in the interlobular septa were centered on blood vessels (angiocentric distribution). Further, the observed lung lesions are likely to be a consequence of ischemia secondary to the vasculitis.

Infectious agents have been implicated in the pathogenesis of vasculitides in both medical (22, 23) and veterinary (24) fields. In mycoplasma infections, vasculitis as a pathological manifestation of disease has been reported previously for Mycoplasma pneumoniae (25), M. synoviae (26), M. mycoides subsp. *capri* (27), M. bovis (28), and M. capricolum subsp. capripneumoniae (29). In a study to determine the efficacy of an inactivated ISCOM vaccine consisting mainly of M. mycoides subsp. mycoides cell membrane proteins, Hübschle et al. (13) reported the presence of pronounced vasculitis in affected lung tissue of vaccinated cattle. Similar to the findings of the present study, vasculitis was not observed in the unvaccinated group. Taken together, these findings suggest that while vasculitis is a feature of M. mycoides subsp. mycoides infections, previous exposure to LppQ-N′ results in an enhanced inflammatory reaction upon experimental infection with M. mycoides subsp. mycoides. This is likely to be associated with the formation of immune complexes, which are fundamental to the development of vasculitis. Hence, rather than providing protection, antibodies to LppQ-N′ may accentuate the disease through enhancement of the vasculitis that is characteristic of infection.

The presence of lesions in the kidneys is typically observed in a proportion of cattle infected with CBPP and has been reported in both field (30) and experimental (31) infections. Kidney lesions were also observed in 88 (12.2%) of 716 cattle killed after an outbreak of CBPP in 61 herds between 1990 and 1991 in Lombardy, Italy (32). Although no attempt was made to isolate live M. mycoides subsp. mycoides from kidneys in the present study, previous isolation of viable M. mycoides subsp. mycoides from kidneys has been achieved (31–33). In two of these studies, the presence of viable M. mycoides subsp. mycoides in the kidney was frequently associated with kidney spots (31, 32). Additionally, it has been demonstrated that M. mycoides subsp. mycoides antigen can be detected in the kidney, even after the clearance of viable M. mycoides subsp. mycoides (34). In a study by Grieco et al., immunohistochemical analysis revealed the presence of M. mycoides subsp. mycoides antigen in the kidneys of 84.4% of cattle with kidney lesions, with some lesions being surrounded by dense lymphoinflammatory infiltrates (32). The current data on vasculitis secondary to CBPP infection and vaccination suggest that the renal necrosis has an ischemic basis.

The reaction in the renal cortex is consistent with deposition of antigen-antibody complexes followed by recruitment of proinflammatory cells to affected areas. The thickening of the glomerular basement membrane and the formation of adhesions to the Bowman's capsule are consistent with immune complex-mediated glomerulonephritis. The pathogenesis of this condition involves penetration of immune complexes across the vascular endothelium but not the basement membrane, resulting in deposition on the endothelial surface. Alternatively, immune complexes may accumulate in the mesangial region of the glomeruli and manifest grossly as thickening of the mesangial basement membrane. Further support for immune complex deposition is given by Grieco et al. (32), who, in acute CBPP infections, observed antigen in the glomeruli as forming a curvilinear pattern in the capillary tuft when immunolabeled. This pattern is consistent with immune complex deposition (35). The lymphocytic aggregates observed in the present case were also observed by Grieco et al. (32) and may arise from the ability of the tubular epithelial cells to act as antigen-presenting cells since they can express major histocompatibility complex (MHC) class II molecules on their surfaces (36).

The observations in the current study have strong parallels with those for another bacterial disease in which an adverse outcome has been observed following vaccination and challenge (37, 38). Van den Bosch and Frey (38) reported that vaccination of pigs with PalA, an outer membrane protein of Actinobacillus pleuropneumoniae, the agent of porcine pleuropneumonia, resulted in enhanced disease rather than protection after experimental infection. In that study, three pigs were vaccinated intramuscularly and boosted with recombinant PalA before challenge with A. pleuropneumoniae aerosol. All three died from the challenge infection, with only one death observed in the control group. Significantly, when PalA was mixed with two other antigens with an established history of protection, the beneficial effects of these antigens was lost. The mechanism for this exacerbated disease in vaccinated pigs remains unclear. Nonetheless, pleural inflammation in porcine pleuropneumonia closely resembles that of CBPP, and it is tempting to speculate that vaccination of cattle with LppQ-N′ precipitates a reaction similar to that observed with PalA in pigs. Any similarities in the enhanced disease induced by prior exposure to PalA and LppQ remain to be determined.

Aside from subunit vaccine experiments, CBPP vaccine research has focused mainly on either live attenuated M. mycoides subsp. mycoides (7, 39, 40) or inactivated formulations of M. mycoides subsp. mycoides surface or total antigens (41–43). In general, the results of these studies suggest that inactivated or whole M. mycoides subsp. mycoides cell lysates induce poor protection, although Gray et al. (42) claimed to have achieved complete protection against natural infection by vaccination with an extreme regimen involving two immunizations with massive doses of M. mycoides subsp. mycoides lysate formulated in complete Freund's adjuvant. While a variety of factors could be responsible for the poor protection induced by these formulations, the results of the current study suggest that determinants such as LppQ-N′ may prime the immune system for potentially harmful responses that are recalled by challenge. The findings of Gray et al. (42) are significant in that massive doses of M. mycoides subsp. mycoides lysate appear to be capable of inducing total protection. This suggests that one or more M. mycoides subsp. mycoides antigens in the lysate induced protective mechanisms that overcame the negative responses provoked by antigens such as LppQ-N′. Therefore, the challenge for CBPP vaccine development remains the identification of antigens that induce the desired protective response. As more antigens are identified and tested for protective capacity, it will be important to demonstrate that they induce protective immune responses rather than predisposing cattle to exacerbated disease.

We have described exacerbation of CBPP pathology following subunit vaccination and subsequent experimental infection with M. mycoides subsp. mycoides and have provided evidence that this exacerbation is associated with immune complex formation. Thus, vaccination of cattle with recombinant LppQ-N′ and experimental challenge with live infective M. mycoides subsp. mycoides resulted in more severe disease that was characterized by vasculitis and glomerulonephritis, both of which are strongly suggestive of immune complex disease. Whereas immune complex disease seems to be a feature of both natural and experimental CBPP, our results indicate that previous exposure to LppQ-N′ enhances this pathological mechanism. These findings support a case for the omission of LppQ-N′ in future vaccines against CBPP and, perhaps, its orthologues in vaccines for other mycoplasma infections.

## References

[1] Amanfu W 2009 Contagious bovine pleuropneumonia (lung sickness) in Africa. Onderstepoort J Vet Res 76:13–17. 19967923

[2] Cottew GS, Yeats FR 1978 Subdivision of Mycoplasma mycoides *subsp*. mycoides from cattle and goats into two types Aust Vet J 54:293–296. 356841 10.1111/j.1751-0813.1978.tb02462.x

[3] Masiga WN, Domenech J, Windsor RS 1996 Manifestation and epidemiology of contagious bovine pleuropneumonia in Africa. Rev Sci Tech 15:1283–1308. 9190017 10.20506/rst.15.4.980

[4] Regalla J, Caporale V, Giovannini A, Santini F, Martel JL, Goncalves AP 1996 Manifestation and epidemiology of contagious bovine pleuropneumonia in Europe. Rev Sci Tech 15:1309–1329. 9190018 10.20506/rst.15.4.979

[5] Thomson GR 2005 Bovine pleuropneumonia and poverty: a strategy for addressing the effects of the disease in sub-Saharan Africa. Centre for Tropical Medicine, University of Edinburgh, Edinburgh, United Kingdom.

[6] Windsor RS, Wood A 1998 Contagious bovine pleuropneumonia: the costs of control in central/southern Africa. Ann N Y Acad Sci 849:299–306. doi:10.1111/j.1749-6632.1998.tb11062.x. 9668478

[7] Thiaucourt F, Yaya A, Wesonga H, Huebschle OJ, Tulasne JJ, Provost A 2000 Contagious bovine pleuropneumonia. A reassessment of the efficacy of vaccines used in Africa. Ann N Y Acad Sci 916:71–80. doi:10.1111/j.1749-6632.2000.tb05276.x. 11193704

[8] Dedieu-Engelmann L 2008 Contagious bovine pleuropneumonia: a rationale for the development of a mucosal sub-unit vaccine. Comp Immunol Microbiol Infect Dis 31:227–238. doi:10.1016/j.cimid.2007.07.009. 17706775 PMC7132392

[9] Herbelin A, Ruuth E, Delorme D, Michel-Herbelin C, Praz F 1994 Mycoplasma arginini TUH-14 membrane lipoproteins induce production of interleukin-1, interleukin-6, and tumor necrosis factor alpha by human monocytes. Infect Immun 62:4690–4694. 7927744 10.1128/iai.62.10.4690-4694.1994 PMC303169

[10] Rawadi G, Roman-Roman S, Castedo M, Dutilleul V, Susin S, Marchetti P, Geuskens M, Kroemer G 1996 Effects of Mycoplasma fermentans on the myelomonocytic lineage. Different molecular entities with cytokine-inducing and cytocidal potential. J Immunol 156:670–678. 8543819

[11] Abdo EM, Nicolet J, Frey J 2000 Antigenic and genetic characterization of lipoprotein LppQ from Mycoplasma mycoides subsp. mycoides SC. Clin Diagn Lab Immunol 7:588–595. doi:10.1128/CDLI.7.4.588-595.2000. 10882657 PMC95919

[12] Bruderer U, Regalla J, Abdo el, Huebschle MOJ, Frey J 2002 Serodiagnosis and monitoring of contagious bovine pleuropneumonia (CBPP) with an indirect ELISA based on the specific lipoprotein LppQ of Mycoplasma mycoides subsp. mycoides SC. Vet Microbiol 84:195–205. doi:10.1016/S0378-1135(01)00466-7. 11731172

[13] Hübschle OJ, Tjipura-Zaire G, Abusugra I, di Francesca G, Mettler F, Pini A, Morein B 2003 Experimental field trial with an immunostimulating complex (ISCOM) vaccine against contagious bovine pleuropneumonia. J Vet Med B Infect Dis Vet Public Health 50:298–303. doi:10.1046/j.1439-0450.2003.00659.x. 14629002

[14] Jores J, Nkando I, Sterner-Kock A, Haider W, Poole J, Unger H, Muriuki C, Wesonga H, Taracha EL 2008 Assessment of in vitro interferon-gamma responses from peripheral blood mononuclear cells of cattle infected with Mycoplasma mycoides *ssp*. mycoides small colony type. Vet Immunol Immunopathol 124:192–197. doi:10.1016/j.vetimm.2008.02.019. 18406471

[15] Brown RD, Gourlay RN, Macleod AK 1965 The production of T1 broth culture contagious bovine pleuropneumonia vaccine. Bull Epizoot Dis Afr 13:149–155. 14344194

[16] Litamoi J, Palya VJ, Sylla D, Rweyemamu MM 1996 Quality control testing of contagious bovine pleuroneumonia live attenuated vaccine; standard operating procedures, p 55 FAO Animal Production Health Paper, vol 128 FAO, Rome, Italy.

[17] Villegas P 1998 Titration of biological suspension, p 248–252. *In* Swayne DE, Glisson JR, Jackwood MW, Pearson JE, Reed WM (ed), A laboratory manual for the isolation and identification of avian pathogens, 4th ed American Association of Avian Pathologists Inc., Kennet Square, PA.

[18] Hudson JR, Turner AW 1963 Contagious bovine pleuropneumonia: a comparison of the efficacy of two types of vaccine. Aust Vet J 39:373–385. doi:10.1111/j.1751-0813.1963.tb01608.x.

[19] Goncalves R, Regalla J, Nicolet J, Frey J, Nicholas R, Bashiruddin J, de Santis P, Goncalves AP 1998 Antigen heterogeneity among Mycoplasma mycoides subsp. mycoides SC isolates: discrimination of major surface proteins. Vet Microbiol 63:13–28. 9810618 10.1016/s0378-1135(98)00214-4

[20] Dedieu L, Balcer-Rodrigues V 2006 Viable Mycoplasma mycoides *ssp*. mycoides small colony-mediated depression of the bovine cell responsiveness to the mitogen concanavalin A. Scand J Immunol 64:376–381. doi:10.1111/j.1365-3083.2006.01799.x. 16970677

[21] Provost A, Perreau P, Breard A, Le Goff C, Martel JL, Cottew GS 1987 Contagious bovine pleuropnemonia. Rev Sci Tech 6:625–679. 10.20506/rst.6.3.306 32370343

[22] Lidar M, Lipschitz N, Langevitz P, Shoenfeld Y 2009 The infectious etiology of vasculitis. Autoimmunity 42:432–438. doi:10.1080/08916930802613210. 19811260

[23] Pipitone N, Salvarani C 2008 The role of infectious agents in the pathogenesis of vasculitis. Best Pract Res Clin Rheumatol 22:897–911. doi:10.1016/j.berh.2008.09.009. 19028370 PMC7106215

[24] Laitinen K, Laurila A, Pyhala L, Leinonen M, Saikku P 1997 Chlamydia pneumoniae infection induces inflammatory changes in the aortas of rabbits. Infect Immun 65:4832–4835. 9353072 10.1128/iai.65.11.4832-4835.1997 PMC175693

[25] Narita M 2010 Pathogenesis of extrapulmonary manifestations of Mycoplasma pneumoniae infection with special reference to pneumonia. J Infect Chemother 16:162–169. doi:10.1007/s10156-010-0044-X. 20186455

[26] Senties-Cue G, Shivaprasad HL, Chin RP 2005 Systemic Mycoplasma synoviae infection in broiler chickens. Avian Pathol 34:137–142. doi:10.1080/03079450500058646. 16191695

[27] Rosendal S 1981 Experimental infection of goats, sheep and calves with the large colony type of Mycoplasma mycoides subsp. mycoides. Vet Pathol 18:71–81. 7008331 10.1177/030098588101800108

[28] Lu X, Rosenbusch RF 2004 Endothelial cells from bovine pulmonary microvasculature respond to Mycoplasma bovis preferentially with signals for mononuclear cell transmigration. Microb Pathog 37:253–261. doi:10.1016/j.micpath.2004.08.001. 15519046

[29] Kusiluka LJ, Semuguruka WD, Kazwala RR, Ojeniy B, Friis NF 2000 Demonstration of Mycoplasma capricolum subsp. capripneumoniae and Mycoplasma mycoides subsp mycoides, small colony type in outbreaks of caprine pleuropneumonia in eastern Tanzania. Acta Vet Scand 41:311–319. 11126580 10.1186/BF03549639 PMC7996431

[30] Bygrave A, Moulton JE, Shifrine M 1968 Clinical, serological and pathological findings in an outbreak of contagious bovine pleuropneumonia. Bull Epizoot Dis Afr 16:25. 5693871

[31] Masiga WN, Windsor RS, Read WC 1972 A new mode of spread of contagious bovine pleuropneumonia? Vet Rec 90:247–248. doi:10.1136/vr.90.9.247. 5063239

[32] Grieco V, Boldini M, Luini M, Finazzi M, Mandelli G, Scanziani E 2001 Pathological, immunohistochemical and bacteriological findings in kidneys of cattle with contagious bovine pleuropneumonia (CBPP). J Comp Pathol 124:95–101. doi:10.1053/jcpa.2000.0433. 11222005

[33] Stone SS, Masiga WN, Read WC 1969 Mycoplasma mycoides transplacental transfer in cattle. Res Vet Sci 10:368–372. 4980224

[34] Buxton D, Rae AG, Maley SW, Thomson KM, Livingstone M, Jones GE, Herring AJ 1996 Pathogenesis of Chlamydia psittaci infection in sheep: detection of the organism in a serial study of the lymph node. J Comp Pathol 114:221–230. doi:10.1016/S0021-9975(96)80044-2. 8762580

[35] Churg J 1982 Primary glomerular diseases. Igaku-Shoin, Tokyo, Japan.

[36] Wuthrich RP, Glimcher LH, Yui MA, Jevnikar AM, Dumas SE, Kelley VE 1990 MHC class II, antigen presentation and tumor necrosis factor in renal tubular epithelial cells. Kidney Int 37:783–792. doi:10.1038/ki.1990.46. 2407890

[37] Lyashchenko K, Whelan AO, Greenwald R, Pollock JM, Andersen P, Hewinson RG, Vordermeier HM 2004 Association of tuberculin-boosted antibody responses with pathology and cell-mediated immunity in cattle vaccinated with Mycobacterium bovis BCG and infected with M. bovis. Infect Immun 72:2462–2467. doi:10.1128/IAI.72.5.2462-2467.2004. 15102752 PMC387859

[38] van den Bosch H, Frey J 2003 Interference of outer membrane protein PalA with protective immunity against Actinobacillus pleuropneumoniae infections in vaccinated pigs. Vaccine 21:3601–3607. doi:10.1016/S0264-410X(03)00410-9. 12922088

[39] Hamsten C, Tjipura-Zaire G, McAuliffe L, Huebschle OJ, Scacchia M, Ayling RD, Persson A 2010 Protein-specific analysis of humoral immune responses in a clinical trial for vaccines against contagious bovine pleuropneumonia. Clin Vaccine Immunol 17:853–861. doi:10.1128/CVI.00019-10. 20357055 PMC2863394

[40] Mbulu RS, Tjipura-Zaire G, Lelli R, Frey J, Pilo P, Vilei EM, Mettler F, Nicholas RA, Huebschle OJ 2004 Contagious bovine pleuropneumonia (CBPP) caused by vaccine strain T1/44 of Mycoplasma mycoides subsp. mycoides SC. Vet Microbiol 98:229–234. doi:10.1016/j.vetmic.2003.11.007. 15036531

[41] Garba SA, Ajayi A, Challa LD, Bello MK, Gazama JN, Gimba H, Ngbede J, Bitrus B, Adeoye AO 1986 Field trial of inactivated oil-adjuvant Gladysdale strain vaccine for contagious bovine pleuropneumonia. Vet Rec 119:376–377. doi:10.1136/vr.119.15.376. 3538638

[42] Gray MA, Simam P, Smith GR 1986 Observations on experimental inactivated vaccines for contagious bovine pleuropneumonia. J Hyg (Lond) 97:305–315. doi:10.1017/S0022172400065402. 3782785 PMC2083532

[43] Hubschle OJ, Tjipura-Zaire G, Abusugra I, di Francesca G, Mettler F, Pini A, Morein B 2003 Experimental field trial with an immunostimulating complex (ISCOM) vaccine against contagious bovine pleuropneumonia. J Vet Med B Infect Dis Vet Public Health 50:298–303. doi:10.1046/j.1439-0450.2003.00659.x. 14629002

